# Reassessing Ethnic Differences in Mean BMI and Changes Between 2007 and 2013 in English Children

**DOI:** 10.1002/oby.22091

**Published:** 2017-12-17

**Authors:** Mohammed T. Hudda, Claire M. Nightingale, Angela S. Donin, Christopher G. Owen, Alicja R. Rudnicka, Jonathan C. K. Wells, Harry Rutter, Derek G. Cook, Peter H. Whincup

**Affiliations:** ^1^ Population Health Research Institute, St George's University of London London UK; ^2^ Childhood Nutrition Research Centre, Population, Policy and Practice Programme, UCL Great Ormond Street Institute of Child Health London UK; ^3^ ECOHOST – The Centre for Health and Social Change, London School of Hygiene and Tropical Medicine London UK

## Abstract

**Objective:**

National body fatness (BF) data for English South Asian and Black children use BMI, which provides inaccurate ethnic comparisons. BF levels and time trends in the English National Child Measurement Programme (NCMP) between 2007 and 2013 were assessed by using ethnic‐specific adjusted BMI (aBMI) for South Asian and Black children.

**Methods:**

Analyses were based on 3,195,323 children aged 4 to 5 years and 2,962,673 children aged 10 to 11 years. aBMI values for South Asian and Black children (relating to BF as in White children) were derived independently. Mean aBMI levels and 5‐year aBMI changes were obtained by using linear regression.

**Results:**

In the 2007‐2008 NCMP, mean aBMIs in 10‐ to 11‐year‐old children (boys, girls) were higher in South Asian children (20.1, 19.9 kg/m^2^) and Black girls, but not in Black boys (18.4, 19.2 kg/m^2^) when compared with White children (18.6, 19.0 kg/m^2^; all *P* < 0.001). Mean 5‐year changes (boys, girls) were higher in South Asian children (0.16, 0.32 kg/m^2^ per 5 y; both *P* < 0.001) and Black boys but not girls (0.13, 0.15 kg/m^2^ per 5 y; *P* = 0.01, *P* = 0.41) compared with White children (0.02, 0.11 kg/m^2^ per 5 y). Ethnic differences at 4 to 5 years were similar. Unadjusted BMI showed similar 5‐year changes but different mean BMI patterns.

**Conclusions:**

BF levels were higher in South Asian children than in other groups in 2007 and diverged from those in White children until 2013, a pattern not apparent from unadjusted BMI data.

## Introduction

High levels of body fatness (BF), overweight, and obesity in children represent a major global public health challenge 1. In England, overweight and obesity together (overweight‐obesity), defined by using BMI (in kilograms per meter squared), affect approximately a third of children aged 2 to 15 years 2. Excessive weight in childhood is associated with higher short‐term health risks, including poor psychological health and the development of asthma 3, as well as increased longer‐term risks of type 2 diabetes (T2D), cardiovascular disease (CVD), and adult overweight‐obesity 4, 5, 6. High BF in South Asian and Black children in England is of particular concern because both ethnic groups have high risks of T2D and CVD in adulthood 7, 8, 9, 10 compared with White children; these risks have their origins in childhood 11, 12. But, reliable evidence on patterns and trends over time of childhood BF in these ethnic groups is limited. Key national data sources, notably the National Child Measurement Programme (NCMP), have used BMI to categorize overweight and obesity, using identical BMI thresholds in all ethnic groups that are based on an exclusively White reference population 13, 14. However, the associations between childhood BMI and BF differ by ethnic group; BMI systematically underestimates BF in South Asian children and overestimates BF in Black children 15, 16. We recently developed ethnic‐specific BMI adjustments, which provide adjusted BMI values (aBMI) for English South Asian and Black children and have the same relation to total BF as in White children, in order to provide more valid comparisons of BF differences between ethnic groups 17. In this study, we have used this approach to reassess patterns and changes over time in childhood BF, overweight, and obesity in South Asian, Black, and White children by using NCMP data between 2007 and 2013.

## Methods

### NCMP: study population and data collection

The NCMP is an annual survey of the weights and heights of English primary school children in Reception and Year 6 classes. At the time of the NCMP examination, Reception children are between 4.0 and 5.9 years of age and Year 6 children are between 10.0 and 11.9 years of age. The survey commenced with the 2006‐2007 study and is currently directed by Public Health England; data collection is carried out by Local Authority (LA) public health departments 13. All state primary schools in England (*n* ≈ 17,000) are invited to participate; within participating schools, all relevant pupils are invited to participate on an opt‐out basis. LA public health departments recruit, train, and supervise assessment teams to measure weight and height. Public Health England provides detailed instructions on instrument choices and measurement techniques. Weight is measured to the nearest 0.1 kg, and height with the child's heels together and the head in the Frankfurt plane to the nearest 0.1 cm. BMI is calculated as weight divided by height squared. School record information on name, date of birth, sex, and parentally defined ethnic group is also collected. Data are entered by using the NCMP electronic system and are collated by the Health and Social Care Information Centre.

### Ethnic group

Ethnic groups were based on school records or the child's health records 13 and were defined by using the National Health Service classification 18. For the present report, children identified as “White British,” “White Irish,” or “any other White background” were grouped as “White.” Children identified as “Black African,” “Black Caribbean,” or “any other Black background” were presumed as of African origin and were grouped together as “Black.” Children of “Indian,” “Pakistani,” or “Bangladeshi” origin were grouped together as “South Asian.” Children of “Chinese” or “Asian other” origins were grouped as “Other Asian.” Children of “any other ethnic background” or “mixed ethnicity” were grouped as “Other Ethnicity.” The main ethnic groups for the purpose of this report were White, Black, and South Asian children. Ethnic subgroups (Black African, Black Caribbean, Black Other, Indian, Pakistani, and Bangladeshi children) were also explored in supplementary analyses.

### aBMI values for Black and South Asian children

Ethnic‐specific BMI adjustments for Black and South Asian children aged 4 to 12 years were derived by using pooled data from four previous United Kingdom–based studies (*N* = 1,725 children) that used the deuterium dilution reference method to make accurate body fat assessments in Black, South Asian, and White children; full details are provided in a previous paper 17. BMI adjustments were derived by using regression models that ensured that aBMI reflected the same level of BF in the same way as in White children 17. To obtain robust BMI adjustments across the full age range studied, age group was fitted in 3‐year age groups (4‐6 y, 7‐9 y, and 10‐12 y). BMI was regressed on height‐independent fat mass index (FMI) (kilograms in meters to the fifth power), fitting ethnic group and age group in boys and girls separately. All two‐way interactions were tested and included (when statistically significant) by using a stepwise forward approach 17. For South Asian children, single sex‐specific positive BMI adjustments of +1.12 (95% CI: 0.83‐1.41) and +1.07 (95% CI: 0.8‐1.39) for boys and girls, respectively, were applicable for all age groups and FMI levels. For Black children, BMI adjustments were all negative but were more complex, varying by age group and FMI levels because there were statistically significant interactions between Black African ethnicity and FMI (*P* = 0.004 for boys; *P* = 0.003 for girls) and also between FMI and age group (*P* < 0.0001 for boys and girls). Adjustments varied between −0.13 (boys) and −0.12 (girls) in 10‐ to 12‐year‐olds with low unadjusted BMI values and −5.52 (boys) and −5.06 (girls) in 4‐ to 6‐year‐olds with high unadjusted BMI values 17.

### Data exclusions

Analyses were restricted to the school years between 2007‐2008 (the first year in which ethnic group was coded in at least two‐thirds of children) and 2012‐2013 (the most recent year for which data were available to us). During that period, 6,173,500 children participated in NCMP (3,204,915 aged 4‐5 y, 2,968,585 aged 10‐11 y). We excluded 15,504 children (0.25%) from analyses, including those measured in LA areas with data quality concerns (*n* = 12,726), those outside the study age range (*n* = 2,773), and those with extreme outlier values of weight, height, and BMI obtained from box and whisker plots (*n* = 5). Hence, 6,157,996 children (3,195,323 aged 4‐5 y and 2,962,673 aged 10‐11 y) were included in further analyses.

### Statistical analysis

Average aBMI levels were determined for the first study year (2007‐2008) by regressing aBMI, stratified by age‐sex groups, on ethnic group, age, and the two‐way interaction between year (categorical) and ethnic group. Differences in aBMI levels between ethnic groups (compared with White children) were formally tested by using the Wald test for differences. This process was repeated by using ethnic subgroups to test for heterogeneity within the Black and South Asian ethnic groups. To assess whether changes over time in aBMI in each ethnic group could be treated as linear, mean aBMI and the corresponding 95% CIs were plotted for each year and the overall changes visually assessed for each age‐sex and ethnic group. No appreciable departure from linearity was observed from the graphs; therefore, linear regression models, stratified by age‐sex groups, were used to investigate average changes over time in aBMI in each ethnic group over the 6‐year period. aBMI was regressed against ethnic group, age, and the two‐way interaction between year and ethnic group, with year fitted as a continuous variable. We calculated the average change in aBMI over a 5‐year period and present this as our summary measure of change. The statistical significance of the changes in each ethnic group was determined by using the Wald test at the 5% significance level. Differences in the change in aBMI over time between ethnic groups (compared with White children) were formally tested by using the Wald test. In order to test for heterogeneity within ethnic subgroups, regression models were repeated by replacing the main ethnic group variable (e.g., White, Black, South Asian) with an ethnic subgroup variable (e.g., Indian, Pakistani, Bangladeshi or Black African, Black Caribbean, Black Other). Regression coefficients for the ethnic subgroups were tested for heterogeneity by using Wald tests at the 5% significance level. The prevalences of overweight‐obesity (based on aBMI values and using the United Kingdom 1990 growth reference thresholds 14) were calculated for the 2007‐2008 year for each age‐sex group and for each ethnic group. Logistic regression models were used to investigate the average 5‐year changes in overweight‐obesity prevalence in each ethnic group over the 6‐year period. Analyses of levels and 5‐year changes were repeated by using unadjusted BMI for comparison with aBMI results.

## Results

A total of 3,195,323 children aged 4‐5 years (51% male) and 2,962,673 children aged 10‐11 years (51% male) contributed to the analyses (Supporting Information Table S1). The proportion of participants with unknown ethnic origin declined with improvements in the completeness of ethnic group recording between 2007‐2008 and 2012‐2013; the proportions of children of White, Black African, and Pakistani origin showed increases over the same period.

### Mean aBMI levels in 2007‐2008 and 5‐year changes in aBMI between 2007‐2008 and 2012‐2013 for White, South Asian, and Black children

**Figure 1:**
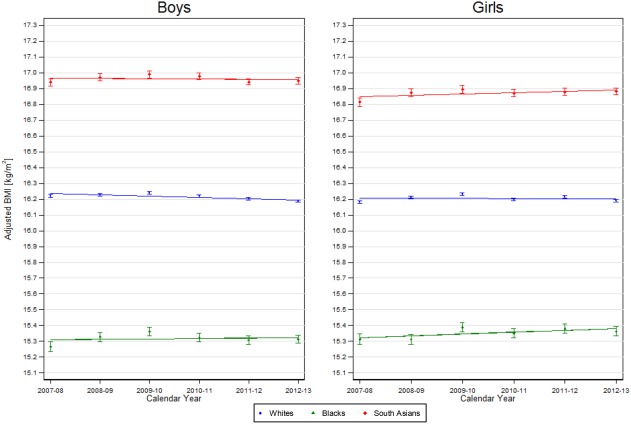
aBMI for 4‐ to 5‐year‐old children in each major ethnic group between 2007‐2008 and 2012‐2013 by sex. Lines represent mean annual change in BMI in each ethnic group across the 6 years from age group and sex‐stratified regression models of aBMI against ethnic group, an interaction between ethnic group and year and age (continuous). Points and corresponding 95% CIs show the mean aBMI level (age adjusted) for each school year.

**Figure 2:**
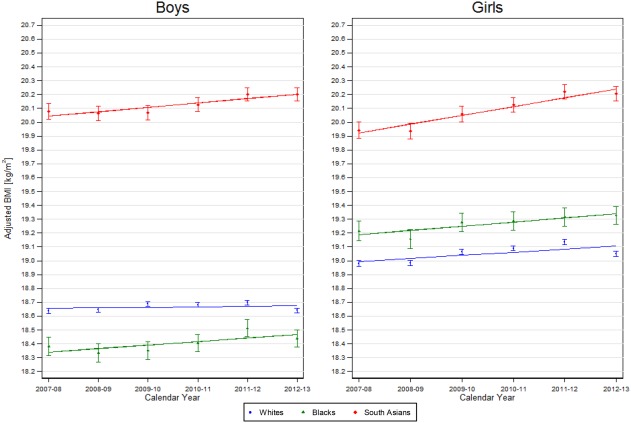
aBMI for 10‐11 year old children in each major ethnic group between 2007‐2008 and 2012‐2013 by sex. Lines represent mean annual change in BMI in each ethnic group across the 6 years from age group and sex stratified regression models of aBMI against ethnic group, an interaction between ethnic group and year and age (continuous). Points and corresponding 95% CIs show the mean aBMI level (age adjusted) for each school year.

Mean aBMI levels in the first year of the study period (2007‐2008) are summarized for each ethnic group in Table 1 for each age‐sex group; 5‐year changes in aBMI between 2007‐2008 and 2012‐2013 (per 5 years) are presented in Table 2. Mean aBMI levels for each ethnic group in each of the six school years are presented for each age‐sex group in Figures 1, 2. Mean aBMI levels in 2007‐2008 and 5‐year changes in aBMI between 2007‐2008 and 2012‐2013 for each ethnic subgroup are summarized in Tables 1, 2, respectively, and also graphically in Supporting Information Figures S1‐S2.

**Table 1:** Mean aBMI (kg/m^2^) in each major ethnic group (top) and ethnic subgroup (bottom) in 2007‐2008 by age group and sex

	4‐5 year olds						10‐11 year olds					
	Males			Females			Males			Females		
	Mean aBMI (kg/m^2^)	SE	*P*	Mean aBMI (kg/m^2^)	SE	*P*	Mean aBMI (kg/m^2^)	SE	*P*	Mean aBMI (kg/m^2^)	SE	*P*
**Ethnic group**												
**White**	16.2	0.00	N/A	16.2	0.01	N/A	18.6	0.01	N/A	19.0	0.01	N/A
**Black**	15.3	0.02	< 0.001	15.3	0.02	< 0.001	18.4	0.03	< 0.001	19.2	0.04	< 0.001
**South Asian**	16.9	0.01	< 0.001	16.8	0.01	< 0.001	20.1	0.03	< 0.001	19.9	0.03	< 0.001
**Ethnic subgroup**												
**Black**												
**African**	15.3	0.02	< 0.001	15.4	0.02	< 0.001	18.4	0.05	< 0.001	19.2	0.05	< 0.001
**Caribbean**	15.2	0.03	< 0.001	15.2	0.03	< 0.001	18.4	0.06	< 0.001	19.2	0.06	< 0.001
**Other**	15.2	0.04	< 0.001	15.2	0.04	< 0.001	18.4	0.09	0.002	19.1	0.10	0.208
**South Asian**												
**Indian**	16.7	0.02	< 0.001	16.6	0.03	< 0.001	19.9	0.05	< 0.001	19.7	0.05	< 0.001
**Pakistani**	17.0	0.02	< 0.001	16.9	0.02	< 0.001	20.1	0.04	< 0.001	20.1	0.05	< 0.001
**Bangladeshi**	17.2	0.03	< 0.001	17.0	0.03	< 0.001	20.5	0.06	< 0.001	20.1	0.07	< 0.001

Age‐adjusted means presented. *P* value for ethnic difference in aBMI from White children.

**Table 2:** Mean 5‐y change in aBMI (kg/m^2^/5 y) in each major ethnic group (top) and ethnic subgroup (bottom) between 2007‐2008 and 2012‐2013 by age group and sex

	4‐5 year olds								10‐11 year olds							
	Males				Females				Males				Females			
	BMI change in 5 y (kg/m^2^)	SE	*P* 1	*P* 2	BMI change in 5 y (kg/m^2^)	SE	*P* 1	*P* 2	BMI change in 5 y (kg/m^2^)	SE	*P* 1	*P* 2	BMI change in 5 y (kg/m^2^)	SE	*P* 1	*P* 2
**Ethnic group**																
**White**	−0.04	0.00	< 0.001	N/A	0.00	0.01	0.556	N/A	0.02	0.01	0.064	N/A	0.11	0.01	< 0.001	N/A
**Black**	0.01	0.02	0.455	0.002	0.06	0.02	0.003	0.002	0.13	0.04	0.001	0.008	0.15	0.04	< 0.001	0.407
**South Asian**	−0.01	0.01	0.425	0.025	0.04	0.02	0.006	0.005	0.16	0.03	< 0.001	< 0.001	0.32	0.03	< 0.001	< 0.001
**Ethnic subgroup**																
**Black**																
**African**	0.03	0.02	0.138	< 0.001	0.05	0.02	0.048	0.040	0.15	0.05	0.004	0.015	0.18	0.06	0.001	0.242
**Caribbean**	−0.07	0.03	0.048	0.457	0.05	0.04	0.211	0.186	0.07	0.07	0.315	0.470	0.16	0.08	0.041	0.571
**Other**	−0.01	0.04	0.764	0.456	0.03	0.05	0.550	0.507	0.17	0.10	0.072	0.114	0.12	0.10	0.231	0.946
**South Asian**																
**Indian**	0.02	0.02	0.381	0.009	0.06	0.03	0.036	0.030	0.13	0.05	0.017	0.046	0.20	0.06	< 0.001	0.168
**Pakistani**	0.01	0.02	0.453	0.004	0.05	0.02	0.014	0.011	0.17	0.05	< 0.001	0.002	0.29	0.05	< 0.001	< 0.001
**Bangladeshi**	−0.08	0.03	0.012	0.273	0.03	0.03	0.320	0.282	0.17	0.07	0.014	0.032	0.44	0.07	< 0.001	< 0.001

Mean 5‐y changes in aBMI were obtained from age group– and sex‐stratified regression models that adjusted for age (continuous). *P* 1 denotes *P* value from testing whether 5‐y changes in aBMI are different from zero in each ethnic group. *P* 2 denotes *P* value from testing whether 5‐y changes in each ethnic group are different from respective 5‐y changes in White children.

#### Four‐ to five‐year‐olds

In 2007‐2008, the mean aBMI level in White boys and girls aged 4‐5 years was 16.2. In comparison with White children, mean aBMI levels were higher in South Asian children (boys: 16.9, girls: 16.8) and lower in Black children (boys and girls: 15.3) (all *P* < 0.001) (Table 1). There was evidence of heterogeneity in aBMI levels among the three South Asian subgroups (both boys and girls, *P* < 0.001), with the highest aBMI levels among Bangladeshi children and the lowest among Indian children. There was also evidence of heterogeneity among the three Black ethnic subgroups (both boys and girls, *P* < 0.001), with the highest aBMI levels among African children. Five‐year changes in mean aBMI between 2007‐2008 and 2012‐2013 were negative for White boys but null for White girls (boys: −0.04, girls: 0.00; *P* < 0.001 and *P* = 0.56, respectively). Corresponding five‐year changes in aBMI in both South Asian and Black girls were positive (0.06 and 0.04, respectively), more so than in White children (*P* = 0.002 and *P* = 0.005, respectively) (Table 2). In both South Asian and Black boys, changes were close to null but were more positive than in White children (*P* = 0.002 and *P* = 0.025, respectively). Five‐year changes in aBMI within the ethnic subgroups (Table 2, Supporting Information Figures S1‐S2) showed no evidence of heterogeneity, except among 4‐ to 5‐year‐old South Asian boys (*P* = 0.02); in this group, changes in Bangladeshi boys appeared negative (indicating a decline in aBMI) compared with Indian and Pakistani boys.

#### Ten‐ to eleven‐year‐olds

In 2007‐2008, mean aBMI levels in White boys and girls aged 10‐11 years were 18.6 and 19.0, respectively. In comparison with White children, mean aBMI levels were higher in South Asian children (boys: 20.1, girls: 19.9) and in Black girls (19.2) but lower in Black boys (18.4) (all *P* < 0.001) (Table 1). There was evidence of heterogeneity in aBMI levels among the three South Asian subgroups (both boys and girls, *P* < 0.001), with higher levels among Bangladeshi and Pakistani children than Indian children but not among Black subgroups. Five‐year changes in mean aBMI between 2007‐2008 and 2012‐2013 were strongly positive for White girls but null for boys (boys: 0.02 kg/m^2^ per 5 y, girls: 0.11 kg/m^2^ per 5 y; *P* = 0.06 and *P* < 0.001, respectively) (Table 2). In comparison with Whites, corresponding 5‐year changes in aBMI in South Asian children were strongly positive, especially in girls (boys: 0.16 kg/m^2^ per 5 y: girls: 0.32 kg/m^2^ per 5 y; both *P* < 0.001). Black children also had positive changes (boys: 0.13 kg/m^2^ per 5 y, girls: 0.15 kg/m^2^ per 5 y) (Table 2), stronger than those in White children for boys but not girls (*P* = 0.008 and *P* = 0.41, respectively). Five‐year changes in aBMI within the ethnic subgroups (Table 2, Supporting Information Figures S1‐S2) showed no evidence of heterogeneity, except among 4‐ to 5‐year‐old South Asian boys; in this group, 5‐year changes in Bangladeshi boys appeared more negative than those in Indian and Pakistani boys.

### Overweight‐obesity prevalence in 2007‐2008 and 5‐year changes between 2007‐2008 and 2012‐2013 for White, South Asian, and Black children

Patterns and changes in overweight‐obesity prevalence based on aBMI were very consistent with patterns and changes in mean aBMI. The United Kingdom 1990 growth reference thresholds were used to categorize children by overweight and obesity status.

#### Four‐ to five‐year‐olds

The prevalences of overweight‐obesity in White children for 2007‐2008 were 24.0% and 21.1% for boys and girls, respectively (Supporting Information Table S2). Corresponding prevalences were appreciably higher in South Asian children (boys: 39.3%, girls: 33.7%) and lower in Black children (boys: 10.2%, girls: 12.2%) than in White children (all *P* < 0.001). The 5‐year changes in overweight‐obesity prevalence were negative in White boys and less strongly negative in White girls. South Asian girls, however, had a strong positive trend, whereas South Asian boys had a weak negative trend; both differed appreciably from White children (Supporting Information Table S3). Changes in Black children did not differ from those in White children.

#### Ten‐ to eleven‐year‐olds

The prevalences of overweight‐obesity in White children for 2007‐2008 were 32.8% and 29.6% for boys and girls respectively (Supporting Information Table S2). Corresponding prevalences were higher in South Asian children (boys: 49.6%, girls: 40.1%; both *P* < 0.001) and in Black girls but not in Black boys (boys: 31.5%, girls: 33.4%; *P* = 0.12, *P* < 0.001). The 5‐year changes in overweight‐obesity prevalence between 2007‐2008 and 2012‐2013 were strongly positive in White girls but close to null in White boys. Both South Asian boys and girls showed positive changes in overweight‐obesity prevalence, greater in girls, which were stronger than those in White children (Supporting Information Table S3). Black children also showed positive changes in overweight‐obesity prevalence, which were stronger than in White children for boys.

### Mean unadjusted BMI levels in 2007‐2008 and 5‐year changes in aBMI between 2007‐2008 and 2012‐2013 for White, South Asian, and Black children

Analyses of mean BMI levels and 5‐year changes were repeated by using unadjusted BMI (Supporting Information Tables S4‐S5). Changes in unadjusted BMI between 2007‐2008 and 2012‐2013 in White, South Asian, and Black children were not materially different from those using aBMI. However, differences were found in the patterns of mean BMI levels in 2007‐2008 in different ethnic groups from those observed with aBMI. In particular, Black children had the highest mean BMI levels in all age‐sex groups. At 4‐5 years, South Asian children had the lowest mean BMI levels; at 10‐11 years, South Asian boys had higher mean BMI and South Asian girls had lower mean BMI than White children, though these mean BMI levels were lower compared with Black children.

## Discussion

We utilized a novel approach, using aBMI values that related similarly to BF in White, South Asian, and Black children 17 to better assess patterns and changes in average aBMI levels and overweight‐obesity prevalences in these ethnic groups in the NCMP between 2007‐2008 and 2012‐2013. The results presented in this report based on aBMI emphasize that the burdens of high BMI levels (and overweight‐obesity prevalences) among South Asian children in the United Kingdom were already considerably higher than those of both White and Black children in 2007‐2008 and increased further between 2007‐2008 and 2012‐2013, particularly among 10‐ to 11‐year‐olds. These patterns were not observed in unadjusted data, which would have given the impression that, although average BMI levels were increasing in the majority of South Asian children, they were still lower (with the exception of older boys) than those in White children.

### Consistency with previous reports

The higher aBMI levels (and in turn, overweight‐obesity prevalences) among South Asian children in 2007‐2008, compared with Whites, at both 4‐5 and 10‐11 years are consistent with the results of other comparative studies that used more direct measures of BF than BMI, including bioimpedance and dual‐energy x‐ray absorptiometry, and observed higher BF in South Asian participants compared with White participants 15, 16, 19, 20. The lower aBMI levels observed in all Black children (except older girls) in 2007‐2008 are also consistent with the results of earlier studies using more direct BF measures, which all showed lower BF in Black participants compared with White participants 15, 16, 20. In the present study, 5‐year changes between 2007‐2008 and 2012‐2013 were similar for adjusted and unadjusted BMI levels; these changes are broadly consistent with those in a previous NCMP report quantifying ethnic trends in obesity prevalence over a similar period 21. Although that earlier report grouped all Asian children together (rather than focusing on South Asian children), it concluded that, among 4‐ to 5‐year‐olds, a weak negative trend was present among White children, with a positive trend in Asian girls. It also reported positive trends in obesity prevalence over time among 10‐ to 11‐year‐old White, Black, and Asian children, which are findings also consistent with those of the present report. Our findings are also at least partly consistent with the results of a report examining ethnic variations in overweight and obesity over time in the Health Survey for England between 1998 and 2009 22, which showed that overweight and obesity prevalences, though declining in White children, were not decreasing among ethnic minority groups, with some (particularly Black Caribbean children in that report) still tending to increase.

### Strengths and limitations

The NCMP is a large‐scale national survey with standardized data collection and quality control procedures. It also benefits from high rates of participation both by state schools and by individuals. In all, ∼93% of all English children of primary school age (4‐11 y) attend state primary schools, making these schools highly representative of all primary school–aged children. Although the remaining children, who attend private primary schools, tend to be more socioeconomically advantaged than those who do not, they account for a small proportion of all children (∼7%). Furthermore, an average of 91% of all eligible children in state primary schools took part in the survey over the 6‐year period. No information is collected on the small proportion of children who opt out of the survey, and therefore the characteristics of these individuals cannot be compared to those who took part in the survey. However, only if the ethnic differences in nonparticipant children differed from those in participants (which appears unlikely) would this materially affect our results. We were able to use six school years of data from 2007‐2008 through to 2012‐2013, with ethnic group coding completed for at least 67% of participants in 2007‐2008, rising to 86% by 2012‐2013. The change in coding rate during the study raises the possibility that differential selection biases may have operated over the analysis period. Yet sensitivity analyses excluding the first year (2007‐2008) had no material effect on the results, and analyses examining changes in the earlier and later years separately did not show large differences. Patterns observed within ethnic subgroups broadly followed the patterns seen within the main ethnic groups. The BMI adjustments used were derived in an independent population by using the reference deuterium dilution method 23 to obtain fat mass estimates based on a pooled resource of 1,725 Black, South Asian, and White children drawn from four separate studies. The use of this pooled data resource in 4‐ to 12‐year‐olds to derive adjustments in three 3‐year age groups provided robust estimates of BMI adjustment factors across the age range studied. Furthermore, the distributions of BMI within the South Asian, Black, and White ethnic groups in the studies used to derive BMI adjustments were very similar to those of the children in the NCMP populations used in this report, suggesting that their application to NCMP data were reasonable. Adjustments were available for South Asian and Black children, who accounted for 54% of ethnic minority participants across the 6 years of NCMP data. However, similar adjustments are not so far available for other ethnic minority groups (particularly other Asian and mixed ethnic‐origin groups) for whom BMI may well provide misleading indications of BF.

### Implications

Our results suggest that the use of aBMI values affects the assessment of BMI levels and estimates of overweight‐obesity prevalence, rather than trends over time. However, the public health implications of BMI trends and levels are interdependent. On the basis of unadjusted data, it would have appeared that levels in South Asian children were low but were increasing toward those of White children. Yet the adjusted results emphasize that the burdens of high aBMI levels (including overweight and obesity) among South Asian children in the United Kingdom were already higher than those of both White and Black children in 2007‐2008 and increased further between 2007‐2008 and 2012‐2013, particularly among 10‐ to 11‐year‐olds. This is of particular concern, given the high long‐term risks of overweight‐obesity, T2D, and CVD in South Asian people 7, 8 from childhood 11, 12. The increasing aBMI levels among South Asian children could indicate increasing levels of BF, which may lead to a further increase in T2D risk in this ethnic group. Moreover, the increasing divergence of BF between South Asian and White children could lead to an increase in the South Asian‐White difference in T2D risk. A further concern is the elevated mean aBMI level in older Black girls, which tended to increase over the 6‐year period between 2007‐2008 and 2012‐2013. This could strengthen the existing tendency to high overweight‐obesity prevalence in young adult Black women in the United Kingdom 7 and reinforce the higher risks of T2D among Black adults in the United Kingdom 8, 9. The size of the BMI differences observed are sufficiently large to be of substantial public health importance. For example, older South Asian children had mean BMI values of ∼1 kg/m^2^ or more, higher than the values in White children in 2007‐2008 and increasing further by 2012‐2013. Such differences, if maintained into adult life, could account for appreciably higher risks of T2D 24 and CVD 6, 25 among South Asian people.

## Conclusion

Analyses using aBMI to reassess BF levels in children in the United Kingdom emphasize the high and increasing burden of high BF in South Asian children, both at 4‐5 years and especially at 10‐11 years, and among older Black girls. Unadjusted BMI does not adequately describe these patterns, and it tends to underestimate BF in South Asian children and overestimate BF in Black children. These findings emphasize the particular need for early overweight‐obesity prevention in South Asian children, in whom the burdens of high BF are very high.

## Supporting information

Supporting Information 1

## References

[1] World Health Organization . Childhood overweight and obesity. www.who.int/dietphysicalactivity/childhood/en/. Accessed February 2017.

[2] Bridges S , Darton R , Evans‐Lacko S , et al.; Joint Health Surveys Unit, NatCen Social Research, UCL. Health Survey for England 2014. Leeds, UK: Health and Social Care Information Centre; 2015.

[3] Reilly JJ , Methven E , McDowell ZC , et al. Health consequences of obesity. Arch Dis Child 2003;88:748‐752. 12937090 10.1136/adc.88.9.748 PMC1719633

[4] Cole TJ , Bellizzi MC , Flegal KM , Dietz WH . Establishing a standard definition for child overweight and obesity worldwide: international survey. BMJ 2000;320:1240‐1243. 10797032 10.1136/bmj.320.7244.1240 PMC27365

[5] Tirosh A , Shai I , Afek A , et al. Adolescent BMI trajectory and risk of diabetes versus coronary disease. N Engl J Med 2011;364:1315‐1325. 21470009 10.1056/NEJMoa1006992 PMC4939259

[6] Owen CG , Whincup PH , Orfei L , et al. Is body mass index before middle age related to coronary heart disease risk in later life? Evidence from observational studies. Int J Obes (Lond) 2009;33:866‐877. 19506565 10.1038/ijo.2009.102 PMC2726133

[7] The Health and Social Care Information Centre . Health Survey for England 2004: the health of ethnic minorities. NHS Digital website. http://content.digital.nhs.uk/catalogue/PUB01209/heal-surv-hea-eth-min-hea-tab-eng-2004-rep.pdf. Published April 21, 2006. Accessed February 2017.

[8] Tillin T , Hughes AD , Godsland IF , et al. Insulin resistance and truncal obesity as important determinants of the greater incidence of diabetes in Indian Asians and African Caribbeans compared with Europeans: the Southall And Brent REvisited (SABRE) cohort. Diabetes Care 2013;36:383‐393. 22966089 10.2337/dc12-0544 PMC3554271

[9] Tillin T , Hughes AD , Mayet J , et al. The relationship between metabolic risk factors and incident cardiovascular disease in Europeans, South Asians, and African Caribbeans: SABRE (Southall and Brent Revisited)—a prospective population‐based study. J Am Coll Cardiol 2013;61:1777‐1786. 23500273 10.1016/j.jacc.2012.12.046 PMC3677086

[10] Wild SH , Fischbacher C , Brock A , Griffiths C , Bhopal R . Mortality from all causes and circulatory disease by country of birth in England and Wales 2001‐2003. J Public Health (Oxf) 2007;29:191‐198. 17456532 10.1093/pubmed/fdm010

[11] Whincup PH , Nightingale CM , Owen CG , et al. Early emergence of ethnic differences in type 2 diabetes precursors in the UK: the Child Heart and Health Study in England (CHASE Study). PLoS Med 2010;7:e1000263. doi:10.1371/journal.pmed.1000263 20421924 10.1371/journal.pmed.1000263 PMC2857652

[12] Whincup PH , Gilg JA , Papacosta O , et al. Early evidence of ethnic differences in cardiovascular risk: cross sectional comparison of British South Asian and white children. BMJ 2002;324:635. 11895820 10.1136/bmj.324.7338.635 PMC84394

[13] Public Health England . National Child Measurement Programme Operational Guidance 2017. https://www.gov.uk/government/uploads/system/uploads/attachment_data/file/377902/NCMP_operational_guidance.pdf. Published 2016. Accessed December 2016.

[14] Cole TJ , Freeman JV , Preece MA . Body mass index reference curves for the UK, 1990. Arch Dis Child 1995;73:25‐29. 7639544 10.1136/adc.73.1.25 PMC1511150

[15] Nightingale CM , Rudnicka AR , Owen CG , Cook DG , Whincup PH . Patterns of body size and adiposity among UK children of South Asian, black African‐Caribbean and white European origin: Child Heart And health Study in England (CHASE Study). Int J Epidemiol 2011;40:33‐44. 21044977 10.1093/ije/dyq180 PMC3043281

[16] Nightingale CM , Rudnicka AR , Owen CG , et al. Are ethnic and gender specific equations needed to derive fat free mass from bioelectrical impedance in children of South Asian, black African‐Caribbean and white European origin? Results of the assessment of body composition in children study. PLoS One 2013;8:e76426. doi: 10.1371/journal.pone.0076426 24204625 10.1371/journal.pone.0076426 PMC3799736

[17] Hudda MT , Nightingale CM , Donin AS , et al. Body mass index adjustments to increase the validity of body fatness assessment in UK Black African and South Asian children. Int J Obes (Lond) 2017;41:1048‐1055. 28325931 10.1038/ijo.2017.75 PMC5500188

[18] The Health and Social Care Information Centre . Ethnic category code 2016. NHS website. http://www.datadictionary.nhs.uk/data_dictionary/attributes/e/end/ethnic_category_code_de.asp?shownav=1. Updated November 29, 2017. Accessed February 2017.

[19] Lee S , Bountziouka V , Lum S , et al. Ethnic variability in body size, proportions and composition in children aged 5 to 11 years: is ethnic‐specific calibration of bioelectrical impedance required? PLoS One 2014;9:e113883. doi:10.1371/journal.pone.011388 25478928 10.1371/journal.pone.0113883 PMC4257615

[20] Shaw NJ , Crabtree NJ , Kibirige MS , Fordham JN . Ethnic and gender differences in body fat in British schoolchildren as measured by DXA. Arch Dis Child 2007;92:872‐875. 17522163 10.1136/adc.2007.117911 PMC2083240

[21] Dinsdale H , Hancock C , Rutter H ; Public Health England . National Child Measurement Programme: Changes in Children's BMI Between 2006/07 and 2012/13. http://webarchive.nationalarchives.gov.uk/20170110170210/https://www.noo.org.uk/NCMP/National_report. Published November 2014. Accessed January 2017.

[22] Karlsen S , Morris S , Kinra S , Vallejo‐Torres L , Viner RM . Ethnic variations in overweight and obesity among children over time: findings from analyses of the Health Surveys for England 1998‐2009. Pediatr Obes 2014;9:186‐196. 23554401 10.1111/j.2047-6310.2013.00159.x PMC4171811

[23] Wells JC , Fewtrell MS . Measuring body composition. Arch Dis Child 2006;91:612‐617. 16790722 10.1136/adc.2005.085522 PMC2082845

[24] Corbin LJ , Richmond RC , Wade KH , et al. BMI as a modifiable risk factor for type 2 diabetes: refining and understanding causal estimates using Mendelian randomization. Diabetes 2016;65:3002‐3007. 27402723 10.2337/db16-0418 PMC5279886

[25] Whitlock G , Lewington S , Sherliker P , et al. Body‐mass index and cause‐specific mortality in 900 000 adults: collaborative analyses of 57 prospective studies. Lancet 2009;373:1083‐1096. 19299006 10.1016/S0140-6736(09)60318-4 PMC2662372

