# Supplementary material for: Reassessing Ethnic Differences in Mean BMI and Changes Between 2007 and 2013 in English Children
Source: Obesity (Silver Spring). 2017 Dec 17;26(2):412–9. doi: 10.1002/oby.22091 (PMC5814928; doi:10.1002/oby.22091)
Supplement: Supplementary file 1 — Supporting Information 1 [file OBY-26-412-s001.docx]

|  | **4-5 Year Old Boys** | | | | | | | | | | | | |
| --- | --- | --- | --- | --- | --- | --- | --- | --- | --- | --- | --- | --- | --- |
| **Ethnicity** | **2007-08** | | | **2008-09** | | **2009-10** | | **2010-11** | | **2011-12** | | **2012-13** | |
|  | **N** | | **%** | **N** | **%** | **N** | **%** | **N** | **%** | **N** | **%** | **N** | **%** |
| Whites | 117703 | 48 | | 144791 | 56 | 157765 | 59 | 163236 | 59 | 173678 | 60 | 197691 | 66 |
| Black - African | 5571 | 2 | | 6275 | 2 | 7235 | 3 | 7551 | 3 | 8514 | 3 | 9003 | 3 |
| Black - Caribbean | 2940 | 1 | | 2730 | 1 | 2872 | 1 | 2833 | 1 | 2916 | 1 | 2838 | 1 |
| Black - Other | 1545 | 1 | | 1913 | 1 | 2296 | 1 | 2606 | 1 | 2897 | 1 | 2627 | 1 |
| Indian | 4959 | 2 | | 5567 | 2 | 6038 | 2 | 6565 | 2 | 7025 | 2 | 7474 | 3 |
| Pakistani | 7329 | 3 | | 8820 | 3 | 9765 | 4 | 10533 | 4 | 10715 | 4 | 11502 | 4 |
| Bangladeshi | 3336 | 1 | | 3689 | 1 | 3690 | 1 | 3927 | 1 | 3805 | 1 | 4215 | 1 |
| Asian Other | 3307 | 1 | | 3627 | 1 | 4411 | 2 | 4695 | 2 | 6304 | 2 | 6184 | 2 |
| Other | 17663 | 7 | | 20179 | 8 | 27823 | 10 | 29102 | 11 | 31943 | 11 | 18832 | 6 |
| Unknown | 79892 | 33 | | 61022 | 24 | 47131 | 18 | 45492 | 16 | 40195 | 14 | 37521 | 13 |
| **Total** | 244245 |  | | 258613 |  | 269026 |  | 276540 |  | 287992 |  | 297887 |  |

SUPPLEMENTARY TABLE S1: Number, %, of children by ethnic group in each age group from each year of data: by sex

|  | **4-5 Year Old Girls** | | | | | | | | | | | | | | |
| --- | --- | --- | --- | --- | --- | --- | --- | --- | --- | --- | --- | --- | --- | --- | --- |
| **Ethnicity** | **2007-08** | | **2008-09** | | **2009-10** | | | **2010-11** | | | **2011-12** | | | **2012-13** | |
|  | **N** | **%** | **N** | **%** | **N** | **%** | | **N** | **%** | | **N** | **%** | | **N** | **%** |
| Whites | 111826 | 48 | 137293 | 56 | 150866 | | 59 | 156119 | | 59 | 165176 | | 60 | 188663 | 66 |
| Black - African | 5471 | 2 | 6180 | 3 | 6979 | | 3 | 7480 | | 3 | 8460 | | 3 | 8520 | 3 |
| Black - Caribbean | 2806 | 1 | 2678 | 1 | 2743 | | 1 | 2826 | | 1 | 2899 | | 1 | 2788 | 1 |
| Black - Other | 1442 | 1 | 1829 | 1 | 2177 | | 1 | 2566 | | 1 | 2750 | | 1 | 2662 | 1 |
| Indian | 4536 | 2 | 5346 | 2 | 5766 | | 2 | 6178 | | 2 | 6667 | | 2 | 7200 | 3 |
| Pakistani | 6878 | 3 | 8476 | 3 | 9400 | | 4 | 10057 | | 4 | 10350 | | 4 | 10855 | 4 |
| Bangladeshi | 3255 | 1 | 3559 | 1 | 3541 | | 1 | 3866 | | 1 | 3659 | | 1 | 4054 | 1 |
| Asian Other | 3056 | 1 | 3740 | 2 | 4293 | | 2 | 4413 | | 2 | 6005 | | 2 | 6062 | 2 |
| Other | 17315 | 7 | 19364 | 8 | 26445 | | 10 | 27499 | | 10 | 30068 | | 11 | 18168 | 6 |
| Unknown | 76173 | 33 | 58372 | 24 | 44828 | | 17 | 43291 | | 16 | 39046 | | 14 | 36040 | 13 |
| **Total** | 232758 |  | 246837 |  | 257038 | |  | 264295 | |  | 275080 | |  | 285012 |  |

|  | **10-11 Year Old Boys** | | | | | | | | | | | |
| --- | --- | --- | --- | --- | --- | --- | --- | --- | --- | --- | --- | --- |
| **Ethnicity** | **2007-08** | | **2008-09** | | **2009-10** | | **2010-11** | | **2011-12** | | **2012-13** | |
|  | **N** | **%** | **N** | **%** | **N** | **%** | **N** | **%** | **N** | **%** | **N** | **%** |
| Whites | 130209 | 51 | 151134 | 59 | 160799 | 63 | 151200 | 60 | 150638 | 60 | 160278 | 64 |
| Black - African | 5345 | 2 | 5561 | 2 | 6060 | 2 | 5971 | 2 | 6027 | 2 | 6172 | 2 |
| Black - Caribbean | 3215 | 1 | 2918 | 1 | 3117 | 1 | 3096 | 1 | 2915 | 1 | 2971 | 1 |
| Black - Other | 1356 | 1 | 1508 | 1 | 1782 | 1 | 2092 | 1 | 2207 | 1 | 2204 | 1 |
| Indian | 4816 | 2 | 5189 | 2 | 5405 | 2 | 5823 | 2 | 5741 | 2 | 5817 | 2 |
| Pakistani | 5979 | 2 | 7455 | 3 | 7894 | 3 | 8517 | 3 | 9394 | 4 | 9675 | 4 |
| Bangladeshi | 2973 | 1 | 3380 | 1 | 3392 | 1 | 3542 | 1 | 3697 | 1 | 3914 | 2 |
| Asian Other | 2588 | 1 | 3402 | 1 | 3491 | 1 | 3743 | 1 | 4223 | 2 | 4325 | 2 |
| Other | 16243 | 6 | 17308 | 7 | 23284 | 9 | 22806 | 9 | 24293 | 10 | 12914 | 5 |
| Unknown | 82578 | 32 | 58483 | 23 | 41624 | 16 | 47216 | 19 | 41052 | 16 | 40513 | 16 |
| **Total** | 255302 |  | 256338 |  | 256848 |  | 254006 |  | 250187 |  | 248783 |  |

|  | **10-11 Year Old Girls** | | | | | | | | | | | |
| --- | --- | --- | --- | --- | --- | --- | --- | --- | --- | --- | --- | --- |
| **Ethnicity** | **2007-08** | | **2008-09** | | **2009-10** | | **2010-11** | | **2011-12** | | **2012-13** | |
|  | **N** | **%** | **N** | **%** | **N** | **%** | **N** | **%** | **N** | **%** | **N** | **%** |
| Whites | 121148 | 50 | 140944 | 58 | 151752 | 62 | 143401 | 59 | 143261 | 60 | 151146 | 64 |
| Black - African | 5291 | 2 | 5422 | 2 | 5946 | 2 | 5847 | 2 | 5963 | 2 | 6154 | 3 |
| Black - Caribbean | 2973 | 1 | 3059 | 1 | 3018 | 1 | 3012 | 1 | 2867 | 1 | 2927 | 1 |
| Black - Other | 1341 | 1 | 1514 | 1 | 1828 | 1 | 1968 | 1 | 2164 | 1 | 2238 | 1 |
| Indian | 4581 | 2 | 4958 | 2 | 4978 | 2 | 5536 | 2 | 5252 | 2 | 5493 | 2 |
| Pakistani | 5655 | 2 | 6783 | 3 | 7488 | 3 | 8179 | 3 | 9107 | 4 | 9322 | 4 |
| Bangladeshi | 2903 | 1 | 3242 | 1 | 3257 | 1 | 3331 | 1 | 3703 | 2 | 3821 | 2 |
| Asian Other | 2445 | 1 | 3158 | 1 | 3362 | 1 | 3620 | 1 | 3996 | 2 | 4175 | 2 |
| Other | 15392 | 6 | 16647 | 7 | 21745 | 9 | 21666 | 9 | 23160 | 10 | 12547 | 5 |
| Unknown | 78390 | 33 | 55615 | 23 | 39644 | 16 | 44787 | 19 | 39331 | 16 | 38756 | 16 |
| **Total** | 240119 |  | 241342 |  | 243018 |  | 241347 |  | 238804 |  | 236579 |  |

SUPPLEMENTARY TABLE S2: PREVALENCE OF OVERWEIGHT-OBESITY* IN EACH MAJOR ETHNIC GROUP (TOP) AND ETHNIC SUBGROUP (BOTTOM) IN 2007-08: BY AGE-GROUP AND SEX

| **Ethnic Group** | | **4-5 Year Olds** | | | | | | **10-11 Year Olds** | | | | | | |
| --- | --- | --- | --- | --- | --- | --- | --- | --- | --- | --- | --- | --- | --- | --- |
|  |  | **Males** | | | **Females** | | | **Males** | | | **Females** | | | |
|  |  | **Prevalence (%)** | **P-Value** | **95% CI** | **Prevalence (%)** | **P-Value** | **95% CI** | **Prevalence (%)** | **P-Value** | **95% CI** | **Prevalence (%)** | **P-Value** | **95% CI** |  |
|  | Whites | 24.0 | N/A | 23.8 , 24.3 | 21.1 | N/A | 20.9 , 21.4 | 32.8 | N/A | 32.6 , 33.1 | 29.6 | N/A | 29.4 , 29.9 |  |
|  | Blacks | 10.2 | <0.001 | 9.7 , 10.9 | 12.2 | <0.001 | 11.5 , 12.9 | 31.5 | 0.116 | 30.6 , 32.4 | 33.4 | <0.001 | 32.5 , 34.3 |  |
|  | South Asian | 39.3 | <0.001 | 38.6 , 40.1 | 33.7 | <0.001 | 32.9 , 34.5 | 49.6 | <0.001 | 48.8 , 50.5 | 40.1 | <0.001 | 39.3 , 41.0 |  |
| **Ethnic Subgroup** | |  |  |  |  |  |  |  |  |  |  |  |  |  |
| Blacks | African | 11.3 | <0.001 | 10.5 , 12.2 | 13.5 | <0.001 | 12.6 , 14.4 | 31.4 | 0.227 | 30.2 , 32.7 | 33.4 | <0.001 | 32.2 , 34.7 |  |
|  | Caribbean | 8.81 | <0.001 | 7.81 , 9.89 | 10.7 | <0.001 | 9.54 , 11.9 | 31.5 | 0.361 | 29.9 , 33.1 | 33.5 | 0.008 | 31.8 , 35.3 |  |
|  | Other | 9.13 | <0.001 | 7.74 , 10.67 | 10.2 | 0.001 | 8.68 , 11.87 | 31.6 | 0.597 | 29.2 , 34.2 | 32.9 | 0.137 | 30.4 , 35.5 |  |
| S.Asians | Indian | 33.9 | <0.001 | 32.6 , 35.2 | 28.3 | <0.001 | 27.0 , 29.7 | 48.2 | <0.001 | 46.8 , 49.7 | 37.4 | <0.001 | 36.0 , 38.8 |  |
|  | Pakistani | 40.6 | <0.001 | 39.4 , 41.7 | 35.4 | <0.001 | 34.2 , 36.5 | 48.8 | <0.001 | 47.6 , 50.1 | 41.3 | <0.001 | 40.0 , 42.6 |  |
|  | Bangladeshi | 44.8 | <0.001 | 43.1 , 46.5 | 37.5 | <0.001 | 35.9 , 39.2 | 53.5 | <0.001 | 51.7 , 55.3 | 42.1 | <0.001 | 40.3 , 43.9 |  |

* Based on adjusted Body Mass Index for Blacks and South Asians

P-Value denotes the p-value from testing if the mean aBMI value is different from the respective value in Whites

SUPPLEMENTARY TABLE S3: MEAN FIVE-YEAR CHANGE IN PREVALENCE OF OVERWEIGHT-OBESITY IN EACH MAJOR ETHNIC GROUP (TOP) AND ETHNIC SUBGROUP (BOTTOM) BETWEEN 2007-08 AND 2012-13: BY AGE-GROUP AND SEX

| **Ethnic Group** | | **4-5 Year Olds** | | | | | | | | | |
| --- | --- | --- | --- | --- | --- | --- | --- | --- | --- | --- | --- |
|  |  |  | **Males** | | | |  | **Females** | | | |
|  |  | **Change in 5years  (%)** | | **95% CI** | **P-Value1** | **P-Value2** | **Change in 5years  (%)** | | **95% CI** | **P-Value1** | **P-Value2** |
|  | Whites | -7.18 | | -8.56 , -5.79 | <0.001 | N/A | -2.79 | | -4.28 , -1.3 | <0.001 | N/A |
|  | Blacks | -4.29 | | -11.07 , 2.57 | 0.219 | 0.415 | 1.86 | | -4.68 , 8.48 | 0.579 | 0.174 |
|  | South Asian | -2.28 | | -5.71 , 1.17 | 0.195 | 0.009 | 5.14 | | 1.47 , 8.84 | 0.006 | <0.001 |
|  |  |  | |  |  |  |  | |  |  |  |
| **Ethnic Subgroup** | |  | |  |  |  |  | |  |  |  |
| Blacks | African | -3.20 | | -11.72 , 5.47 | 0.468 | 0.367 | -0.50 | | -8.71 , 7.85 | 0.906 | 0.592 |
|  | Caribbean | -18.12 | | -33.03 , -2.74 | 0.021 | 0.163 | 6.31 | | -8.01 , 21.04 | 0.391 | 0.217 |
|  | Other | -7.21 | | -23.5 , 9.63 | 0.397 | 0.997 | -2.60 | | -18.6 , 13.93 | 0.754 | 0.981 |
| S.Asians | Indian | 1.04 | | -5.25 , 7.42 | 0.746 | 0.012 | 7.74 | | 0.93 , 14.65 | 0.026 | 0.003 |
|  | Pakistani | 0.72 | | -4.19 , 5.67 | 0.775 | 0.002 | 7.99 | | 2.76 , 13.27 | 0.003 | <0.001 |
|  | Bangladeshi | -10.55 | | -18.03 , -2.95 | 0.007 | 0.391 | -1.54 | | -9.49 , 6.53 | 0.706 | 0.592 |

| **Ethnic Group** | | **10-11 Year Olds** | | | | | | | | | |
| --- | --- | --- | --- | --- | --- | --- | --- | --- | --- | --- | --- |
|  |  |  | **Males** | | | |  | **Females** | | | |
|  |  | **Change in 5years  (%)** | | **95% CI** | **P-Value1** | **P-Value2** | **Change in 5years  (%)** | | **95% CI** | **P-Value1** | **P-Value2** |
|  | Whites | 0.88 | | -0.44 , 2.2 | 0.190 | N/A | 5.54 | | 4.14 , 6.95 | <0.001 | N/A |
|  | Blacks | 7.53 | | 2.58 , 12.53 | 0.003 | 0.011 | 7.23 | | 2.34 , 12.17 | 0.004 | 0.517 |
|  | South Asian | 11.70 | | 7.99 , 15.43 | <0.001 | <0.001 | 16.81 | | 12.92 , 20.73 | <0.001 | <0.001 |
|  |  |  | |  |  |  |  | |  |  |  |
| **Ethnic Subgroup** | |  | |  |  |  |  | |  |  |  |
| Blacks | African | 9.13 | | 2.42 , 15.94 | 0.008 | 0.018 | 8.75 | | 2.11 , 15.47 | 0.010 | 0.357 |
|  | Caribbean | 4.51 | | -4.65 , 13.83 | 0.337 | 0.447 | 7.34 | | -1.74 , 16.58 | 0.114 | 0.706 |
|  | Other | 7.36 | | -4.75 , 19.76 | 0.236 | 0.301 | 6.32 | | -5.65 , 18.57 | 0.304 | 0.903 |
| S.Asians | Indian | 7.71 | | 1.24 , 14.27 | 0.019 | 0.043 | 13.15 | | 6.23 , 20.15 | <0.001 | 0.035 |
|  | Pakistani | 13.88 | | 8.46 , 19.36 | <0.001 | <0.001 | 15.89 | | 10.24 , 21.6 | <0.001 | <0.001 |
|  | Bangladeshi | 13.92 | | 5.72 , 22.24 | <0.001 | 0.002 | 20.13 | | 11.73 , 28.67 | <0.001 | 0.001 |

Overweight-obesity determined using aBMI values

Mean Annual Changes were obtained from age-group and sex stratified logistic regression models which adjusted for age

Change in 5years corresponds to the relative % change from the prevalence in 2007-08

P-Value1 denotes the p-value from testing whether the 5-year changes in prevalence is different from zero in each ethnic group

P-Value2 denotes the p-value from testing whether the 5-year changes in prevalence is different from the respective change in Whites

SUPPLEMENTARY TABLE S4: MEAN **UNADJUSTED** BODY MASS INDEX (KG/M^2^) IN EACH MAJOR ETHNIC GROUP (TOP) AND ETHNIC SUBGROUP (BOTTOM) IN 2007-08: BY AGE-GROUP AND SEX

| **Ethnic Group** | | **4-5 Year Olds** | | | | | | **10-11 Year Olds** | | | | |  |
| --- | --- | --- | --- | --- | --- | --- | --- | --- | --- | --- | --- | --- | --- |
|  |  | **Males** | | | **Females** | | | **Males** | | | **Females** | |  |
|  |  | **Mean BMI (kg/m²)** | **SE** | **P-Value** | **Mean BMI (kg/m²)** | **SE** | **P-Value** | **Mean BMI (kg/m²)** | **SE** | **P-Value** | **Mean BMI (kg/m²)** | **SE** | **P-Value** |
|  | Whites | 16.2 | 0.00 | N/A | 16.2 | 0.01 | N/A | 18.6 | 0.01 | N/A | 19.0 | 0.01 | N/A |
|  | Blacks | 16.4 | 0.02 | <0.001 | 16.5 | 0.02 | <0.001 | 19.4 | 0.03 | <0.001 | 20.3 | 0.04 | <0.001 |
|  | South Asian | 15.8 | 0.01 | <0.001 | 15.7 | 0.01 | <0.001 | 19.0 | 0.03 | <0.001 | 18.9 | 0.03 | <0.001 |
|  | Asian Other | 15.9 | 0.03 | <0.001 | 15.8 | 0.03 | <0.001 | 19.1 | 0.07 | <0.001 | 18.6 | 0.07 | <0.001 |
|  | Other | 16.2 | 0.01 | 0.567 | 16.2 | 0.01 | 0.229 | 19.1 | 0.03 | <0.001 | 19.3 | 0.03 | <0.001 |
|  | Unknown | 16.2 | 0.01 | 0.001 | 16.1 | 0.01 | <0.001 | 18.7 | 0.01 | <0.001 | 19.1 | 0.01 | <0.001 |
| **Ethnic Subgroup** | |  |  |  |  |  |  |  |  |  |  |  |  |
| Blacks | African | 16.5 | 0.02 | <0.001 | 16.6 | 0.02 | <0.001 | 19.4 | 0.05 | <0.001 | 20.3 | 0.05 | <0.001 |
|  | Caribbean | 16.4 | 0.03 | <0.001 | 16.3 | 0.03 | <0.001 | 19.5 | 0.06 | <0.001 | 20.3 | 0.07 | <0.001 |
|  | Other | 16.3 | 0.04 | 0.005 | 16.3 | 0.05 | <0.001 | 19.4 | 0.09 | <0.001 | 20.1 | 0.10 | <0.001 |
| S.Asians | Indian | 15.6 | 0.02 | <0.001 | 15.5 | 0.03 | <0.001 | 18.7 | 0.05 | 0.068 | 18.6 | 0.05 | <0.001 |
|  | Pakistani | 15.9 | 0.02 | <0.001 | 15.8 | 0.02 | <0.001 | 19.0 | 0.04 | <0.001 | 19.0 | 0.05 | 0.407 |
|  | Bangladeshi | 16.0 | 0.03 | <0.001 | 15.9 | 0.03 | <0.001 | 19.3 | 0.06 | <0.001 | 19.0 | 0.07 | 0.627 |

Age adjusted means presented

P-Value for difference in BMI from Whites

SUPPLEMENTARY TABLE S5: MEAN FIVE-YEAR CHANGE IN **UNADJUSTED** BODY MASS INDEX (KG/M^2^/5YEARS) IN EACH MAJOR ETHNIC GROUP (TOP) AND ETHNIC SUBGROUP (BOTTOM) BETWEEN 2007-08 AND 2012-13: BY AGE-GROUP AND SEX

| **Ethnic Group** | | **4-5 Year Olds** | | | | | | | | **10-11 Year Olds** | | | | | | | |
| --- | --- | --- | --- | --- | --- | --- | --- | --- | --- | --- | --- | --- | --- | --- | --- | --- | --- |
|  |  | **Males** | | | | **Females** | | | | **Males** | | | | **Females** | | | |
|  |  | **BMI Change in 5years  (kg/m2)** | **SE** | **P-Value1** | **P-Value2** | **BMI Change in 5years  (kg/m2)** | **SE** | **P-Value1** | **P-Value2** | **BMI Change in 5years  (kg/m2)** | **SE** | **P-Value1** | **P-Value2** | **BMI Change in 5years  (kg/m2)** | **SE** | **P-Value1** | **P-Value2** |
|  | Whites | -0.04 | 0.00 | <0.001 | N/A | 0.00 | 0.01 | 0.577 | N/A | 0.02 | 0.01 | 0.058 | N/A | 0.11 | 0.01 | <0.001 | N/A |
|  | Blacks | 0.02 | 0.02 | 0.237 | <0.001 | 0.07 | 0.02 | <0.001 | <0.001 | 0.13 | 0.04 | <0.001 | 0.007 | 0.15 | 0.04 | <0.001 | 0.362 |
|  | South Asian | -0.01 | 0.01 | 0.430 | 0.027 | 0.04 | 0.02 | 0.006 | 0.006 | 0.16 | 0.03 | <0.001 | <0.001 | 0.32 | 0.03 | <0.001 | <0.001 |
|  | Asian Other | 0.09 | 0.03 | <0.001 | <0.001 | 0.12 | 0.03 | <0.001 | <0.001 | 0.18 | 0.07 | 0.010 | 0.025 | 0.21 | 0.07 | 0.004 | 0.188 |
|  | Other | -0.05 | 0.01 | <0.001 | 0.665 | 0.00 | 0.01 | 0.985 | 0.862 | 0.08 | 0.03 | 0.012 | 0.076 | 0.20 | 0.03 | <0.001 | 0.013 |
|  | Unknown | -0.02 | 0.01 | 0.050 | 0.005 | 0.02 | 0.01 | 0.049 | 0.047 | 0.01 | 0.02 | 0.721 | 0.486 | 0.01 | 0.02 | 0.739 | <0.001 |
| **Ethnic Subgroup** | |  |  |  |  |  |  |  |  |  |  |  |  |  |  |  |  |
| Blacks | African | 0.05 | 0.02 | 0.041 | <0.001 | 0.06 | 0.02 | 0.013 | 0.010 | 0.16 | 0.05 | 0.003 | 0.011 | 0.19 | 0.06 | <0.001 | 0.189 |
|  | Caribbean | -0.08 | 0.04 | 0.018 | 0.253 | 0.06 | 0.04 | 0.116 | 0.102 | 0.07 | 0.07 | 0.349 | 0.517 | 0.16 | 0.08 | 0.036 | 0.535 |
|  | Other | -0.01 | 0.04 | 0.800 | 0.436 | 0.04 | 0.05 | 0.439 | 0.404 | 0.18 | 0.10 | 0.061 | 0.099 | 0.12 | 0.10 | 0.238 | 0.959 |
| S.Asians | Indian | 0.02 | 0.02 | 0.386 | 0.010 | 0.06 | 0.03 | 0.038 | 0.032 | 0.13 | 0.06 | 0.017 | 0.048 | 0.20 | 0.06 | <0.001 | 0.172 |
|  | Pakistani | 0.01 | 0.02 | 0.459 | 0.004 | 0.05 | 0.02 | 0.015 | 0.013 | 0.17 | 0.05 | <0.001 | 0.002 | 0.29 | 0.05 | <0.001 | <0.001 |
|  | Bangladeshi | -0.08 | 0.03 | 0.013 | 0.279 | 0.03 | 0.03 | 0.325 | 0.289 | 0.17 | 0.07 | 0.014 | 0.034 | 0.44 | 0.07 | <0.001 | <0.001 |

Mean 5-year changes in BMI were obtained from age-group and sex stratified regression models which adjusted for age

P-Value1 denotes the p-value from testing whether the 5-year changes in BMI is different from zero in each ethnic group

P-Value2 denotes the p-value from testing whether the 5-year changes in each ethnic group is different from the respective 5-year changes in White

SUPPLEMENTARY FIGURE S1: ADJUSTED BODY MASS INDEX FOR 4-5 YEAR OLD CHILDREN IN EACH ETHNIC SUBGROUP BETWEEN 2007-08 AND 2012-13: BY SEX

Lines represent mean annual change in BMI in each ethnic group across the six years from age-group and sex stratified linear models regressing aBMI against ethnic group, an interaction between ethnic group and year and age

Points and corresponding 95% CI show the mean aBMI level (age-adjusted) for each school year

SUPPLEMENTARY FIGURE S2: ADJUSTED BODY MASS INDEX FOR 10-11 YEAR OLD CHILDREN IN EACH ETHNIC SUBGROUP BETWEEN 2007-08 AND 2012-13: BY SEX

Lines represent mean annual change in BMI in each ethnic group across the six years from age-group and sex stratified linear models regressing aBMI against ethnic group, an interaction between ethnic group and year and age

Points and corresponding 95% CI show the mean aBMI level (age-adjusted) for each school year
